# Human immunodeficiency virus infection and older adults: A retrospective single-site cohort study from Johannesburg, South Africa

**DOI:** 10.4102/sajhivmed.v19i1.838

**Published:** 2018-11-29

**Authors:** India Butler, William MacLeod, Pappie P. Majuba, Brent Tipping

**Affiliations:** 1 Division of Geriatric Medicine, Department of Internal Medicine, School of Clinical Medicine, Faculty of Health Sciences, University of the Witwatersrand, South Africa; 2 Health Economics and Epidemiology Research Unit, School of Clinical Medicine, Faculty of Health Sciences, University of the Witwatersrand, South Africa; 3 Right to Care, Helen Joseph Hospital, South Africa

## Abstract

**Introduction:**

HIV-infected adults aged over 50 years in South Africa are increasing. This study explored differences between baseline characteristics and 12-month outcomes of younger and older HIV-infected adults initiated on antiretroviral therapy (ART). Additionally, associations with outcomes within the older group were sought.

**Methods:**

We retrospectively reviewed treatment-naive HIV-infected adult patients at ART initiation. Patients aged 18.0–39.9 years were compared to patients aged over 50 years using log-binomial regression for baseline characteristics and 12-month outcomes. Within the older group, outcome associations were found using multivariate regression.

**Results:**

The older cohort (*n* = 1635) compared to the younger cohort (*n* = 10726) comprised more males (47.2% vs. 35.4%, PR 1.52, *p* < 0.05), smokers (12.9% vs. 9.7%, PR 1.32, *p* < 0.05) and overweight patients (26.0% vs. 20.0%, PR 1.32, *p* < 0.05). Fewer older patients had tuberculosis (10.2% vs. 15.3%, PR 0.67, *p* < 0.05), other opportunistic infections (16.9% vs. 23.3%, PR 0.70, *p* < 0.05), World Health Organization stage 3/4 disease (39.9% vs. 43.2%, PR 0.89, *p* < 0.05), anaemia (22.8% vs. 28.4%, PR 0.77, *p* < 0.05), liver dysfunction (17.1% vs. 21.3%, PR 0.83, *p* < 0.05) or low CD4+ count < 100 cells/mm^3^ (56.3% vs. 59.9%, PR 0.71, *p* < 0.05).

Mortality was higher in the older cohort (11.3% vs. 7.5%, PR 1.48, *p* < 0.05). Virological suppression was greater in the older cohort (89.5% vs. 86.5%, PR 1.28, *p* < 0.05) but CD4+ restitution was lower (62.8% vs. 75.0%, PR 0.61, *p* < 0.05). There was no difference in treatment complications between the groups.

Within the older cohort, associations with death were as follows: age > 55 years (PR 1.47, *p* < 0.05), an AIDS-defining condition (PR 2.28, *p* < 0.05), raised ALT (PR 1.53, *p* < 0.05) and CD4+ < 100 cells/mm^3^ (PR 2.15, *p* < 0.05). Associations with favourable treatment response at 12 months were unemployment (PR 1.18, *p* < 0.05) and raised ALT (PR 1.19, *p* < 0.05). Associations with a treatment complication at 12 months were unemployment (PR 1.12, *p* < 0.05), smoking (PR 1.20, *p* < 0.05) and nevirapine use (PR 1.36, *p* < 0.05) but secondary education was protective (PR 0.87, *p* < 0.05).

**Conclusion:**

HIV-infected South African adults aged over 50 years differ in characteristics and outcomes compared to their younger counterparts and justify specialised management within HIV treatment facilities.

## Introduction

South Africa is presently home to the largest number of human immunodeficiency virus (HIV)-infected people and the biggest antiretroviral programme in the world.^1^ In 2015, 6.3 million people in South Africa were living with HIV, and of those, 3.1 million people were on antiretroviral treatment (ART) (SA Department of Health Fact Sheet). Individuals infected with HIV and aged 50 years or more are considered ‘older’.^2,3,4,5^ An increasing number of older adults are being diagnosed and living with HIV infection worldwide. In South Africa, the prevalence of HIV infection among older adults remains high. Estimates from 2012 show that the average prevalence in those over 50 years old is 7.6% (95% CI 6.5–8.8).^6^ In the next 30 years, the prevalence of HIV infection in persons aged over 50 years is predicted to double in South Africa, whereas the prevalence in the 15–49 years age group is expected to halve.^7^

Possible reasons for rising HIV prevalence in the older age group in South Africa include increased rate of new infections, improved case finding because of awareness of this dimension of the HIV epidemic, lengthened survival on ART and, lastly, a ‘shift’ of the disease towards older age groups as the incidence in the younger age groups is reduced. Heterosexual transmission is the predominant mode of spread in South Africa.^6,8,9^

Diagnosis of HIV infection may be delayed in the older age group. Lack of education about HIV is a barrier to detection in older adults as they are less likely to voluntarily test for HIV.^10^ HIV education in South Africa does not reach over a third of older adults.^9^ HIV prevention and testing is not targeted towards older people in South Africa, and healthcare care workers may not suspect or test for the illness in this population leading to missed or delayed diagnosis.^11^

‘Immunosenescence’ is the term used for the complex immunological changes associated with human ageing. These changes affect all components of the immune system, and the net effect is that immunity becomes inefficient and dysfunctional. Human immunodeficiency virus infection leads to immunological changes very similar to changes seen in normal ageing. Older HIV-infected patients will have immune deficits from HIV infection that are additive to the weakened immunity of ageing.^12^ Furthermore, immune restoration with ART in older patients is thought to be incomplete or delayed.^13^

Pre-ART international and southern African studies confirm that older age at the time of HIV infection results in faster progression to death.^14,15^ Mortality rates in older adults with HIV on ART in South Africa in a large multicentre cohort study were significantly higher than age-specific mortality rates of the general population.^4^

A large multicohort study conducted in Europe showed that older patients have better or similar virological responses and better adherence to ART but poorer immunological responses compared to younger patients.^16^ South African data confirm this observation. Older age groups were as (or more) likely to achieve viral suppression, but CD4+ increases after initiation of ART were impaired and mortality was increased.^4,11,17^

Treatment of HIV-infected older adults can be complex. Older HIV-infected adults have higher rates of comorbidities when compared to HIV-uninfected adults of the same age.^18^ Patients may be taking multiple non-ART medications with the potential for adverse drug interactions. Physiological changes in body composition and age-related renal and liver dysfunction may alter drug pharmacokinetics and pharmacodynamics. This, and the frequent presence of comorbidities and other medications potentially causing drug-drug interactions, can lead to abnormal handling and increased toxicity of ART.^13,19^ The frequency of HIV treatment drug-related toxicity is increased in older adults with patients aged over 60 years being more likely to require either a switch or discontinuation of treatment because of drug toxicity.^13,16^ HIV-infected older adults are more sensitive to medication side effects.^20^

This study’s objectives were to compare the baseline demographic, clinical, laboratory and ART regimen variables and then the 12-month clinical, virological and immunological outcomes as well as the prevalence of treatment complications in older versus younger HIV-infected patients initiated onto ART. A further objective was to seek any associations with 12-month outcomes within the older cohort only.

## Methods

### Study design and setting

A single-centred retrospective cohort study of HIV-infected and ART-initiated patients attending Themba Lethu Clinic (TLC) in Johannesburg, South Africa, was conducted. TLC is a public sector HIV treatment site in Johannesburg, South Africa. The clinic is located within Helen Joseph Hospital, a tertiary level urban teaching hospital affiliated to the University of the Witwatersrand. TLC was established in 2004 and is administered by the South African Department of Health as a Comprehensive Care, Management and Treatment site. Right to Care is a non-governmental organisation that assists TLC via funding from the United States Agency for International Development (USAID) and the President’s Emergency Plan for AIDS Relief (PEPFAR). TLC had about 30 000 HIV-infected patients enrolled of which about 21 000 had been initiated on ART by 01 October 2011 since inception.^21^

### Inclusion criteria, outcomes and definitions

Study subjects were included if they were HIV-infected non-pregnant adults, ART-naïve at initiation, eligible for initiation and on first-line ART regimens according to standard public sector guidelines. The time frame included patients initiated on ART from 01 January 2004 until 10 October 2011. The study encompassed two treatment guideline periods. If treatment was initiated before 01 April 2010, then patients with CD4+ count of < 200 cells/mm^3^ or World Health Organization (WHO) stage 4 status (irrespective of CD4+ count) were eligible for treatment under the 2004 guideline. If treatment was initiated from 01 April 2010 onwards, then patients with CD4+ < 200 cells/mm^3^ or CD4 < 350 cells/mm^3^ diagnosed with tuberculosis (TB) or WHO stage 4 status (irrespective of CD4+ count) were eligible for treatment under the 2010 guideline. The cohort was divided into two age groups, namely patients aged above 50 years and patients aged between 18 and 39.9 years. Patients aged between 40 and 49.9 years were excluded because of physiological similarities that might confound the results of comparisons based on age.^22^

The following outcome measures after 12 months of ART were used:

- **Status at 12 months:** Alive and in Care (AIC) versus Dead, Lost to Follow-Up (LTFU) or Transferred out (TF out).
- **Treatment response at 12 months (within those that were AIC):** A favourable outcome was defined as both viral load (VL) suppressed (< 400 copies/mL at 12 months) and CD4+ count increased by > 100 cells/mm^3^ from baseline at 12 months versus an unfavourable outcome (one or both of those criteria not being met).
- **Treatment complication at 12 months (within those that were AIC):** The presence of any treatment complication at 12 months (indicated by the following surrogate measures: regimen change, single drug switch, drug toxicity variable noted, VL at 6 months > 400 copies/mL, missed medical appointment, missed drug pickup or low self-reported adherence) versus the absence of any treatment complication.

LTFU was defined as three months late for a scheduled appointment without a later visit. Clinic counsellors attempted to trace patients who were LTFU and some deaths were identified this way. Deaths were further ascertained within the LTFU group by cross checking with the South African Vital Registration records using the patient’s South African national identity (ID) number if known.^23,24^ Patients were seen monthly for the first six months after initiation, then every second month unless there was a clinical reason for more frequent visits. Laboratory monitoring of CD4+ count and VL is done every six months.^23^

Treatment non-naivety at initiation was defined if a variable indicative of treatment-naive status was negative, if baseline VL was suppressed suggestive of prior treatment or in patients not meeting standard criteria for initiation according to the South African National Department of Health Guidelines of that time.

Polypharmacy was defined as more than five non-ART medications. Non-ART drugs associated with toxicity or a drug interaction are listed in the appendices.^20^

### Data collection and statistical analysis

Data were collected and stored on TherapyEdge-HIVTM (version 3.2 for Windows, Topsfield, MA, USA: Therapy Edge Inc. ABL S.A. 2005): a patient management system. The data collected included demographic, laboratory, medication and clinical data as well as clinic visits and drug pickups. The system is linked with the South African death registry if the patient has a valid national ID number. TherapyEdge-HIVTM is an electronic records collections system. Prior to 2007, data were collected on paper and entered onto the system by data capturers; subsequently, clinicians entered data at the time of clinical encounter with the patient.^23^ Data were exported into SAS Software (version 9.3 for Windows, Cary, NC, USA: SAS Institute Inc. 2002–2010) where it was cleaned and the variables of interest were coded. The Therapy Edge Database used in this paper was made possible by the generous support of the American people through cooperative agreement AID-674-A-12-0020 from USAID.

The first part of this study was a comparison of the older cohort to the younger cohort. Comparisons of the demographic, clinical and laboratory variables as well as ART regimens and non-ART medications at the time of initiation (baseline) variables were done using log-binomial regression. Continuous variables were categorised into clinically meaningful categories.

The second part of the study was to compare the prevalence of the following outcomes after 12 months of ART in the different patient groups: status, treatment response and treatment complication presence. The older and younger groups were compared for each of the outcomes, using log-binomial regression.

The third part of the study measured the associations between demographic, clinical and laboratory characteristics as well as ART regimens and non-ART medications at baseline and outcome variables within the older cohort only. For each of the outcomes, the groups were initially compared with each of the independent variables, using log-binomial regression. Subsequently, a multivariate analysis was done adjusting for known confounders identified from existing data: gender, baseline haemoglobin (Hb) and baseline body mass index (BMI).^17^ Before commencing multivariate analysis, bivariate correlation analysis was conducted among the independent variables to explore potential confounding relationships that should be accounted for in the multivariate analysis. Phi coefficients were determined between two dichotomous variables and Cramer’s V between two categorical variables. There were no strongly confounded variables (phi coefficient or Cramer’s V > 0.50).

Data analysis was carried out in SAS Software (version 9.4 for Windows, Cary, NC, USA: SAS Institute Inc. 2002–2010). The 5% significance level was used.

## Ethical consideration

Although TLC has blanket ethical approval for analysis of data from the Human Research Ethics Committee (Medical) of the University of the Witwatersrand, ethical approval for this study was granted by the ethical review committee. Patient information was confidential and data were de-identified.

## Results

### Patient selection

The master data set was created on 10 October 2012. There were 21 536 records within the data time frame range and with age and initiation date variables available. A further 9175 records were excluded as outside the age ranges 18–39.9 years and > 50 years, pregnant, not treatment-naive or not on standard first-line treatment regimens at initiation, leaving 12 361 records for analysis.

### Baseline characteristics

An overview of the baseline demographic, clinical, laboratory and regimen characteristics is provided in Table 1. The median age of the younger cohort was 32.8 years (IQR 29.0–36.1) and of the older cohort was 54.1 years (IQR 51.8–57.6). The older population had a higher proportion of males than the younger population (47.2% vs. 35.4%, *p* < 0.05). Fewer in the older population had a background of secondary education (48.5% vs. 84.5%, *p* < 0.05) but the percentage of those unemployed at the time of initiation was the same. The older population reported more smoking (12.9% vs. 9.7%, *p* < 0.05). A smaller proportion of the older cohort had TB (10.2% vs. 15.3%, *p* < 0.05) or other opportunistic infections (OI) (16.9% vs. 23.3%, *p* < 0.05) at initiation of ART. Fewer older patients were classified as WHO stage 3 or 4 at initiation compared to the younger patients (39.9% vs. 43.2%, *p* < 0.05). A larger proportion of the older cohort was in the overweight category (having a BMI > 25.0 kg/m^2^) (26.0% vs. 20.0%, *p* < 0.05). Fewer older patients were anaemic with Hb < 10 g/dL (22.8% vs. 28.4%, *p* < 0.05) and fewer had raised alanine transaminase (ALT) > 40 U/L as a marker of liver disease (17.1% vs. 21.3%, *p* < 0.05) when compared to the younger group. A smaller proportion of the older cohort had a low baseline CD4+ category of < 100 cells/mm^3^ (56.3% vs. 59.9%, *p* < 0.05). Fewer older patients were initiated on nevirapine (NVP) (3.7% vs. 12.1%, *p* < 0.05) as the non-nucleotide reverse transcriptase inhibitor (NNRTI) component compared to efavirenz (EFV). A larger number were initiated on either zidovudine (ZDV) (5% vs. 3.7%, *p* < 0.05) or tenofovir (TDF) (22.1% vs. 19.6%, *p* < 0.05) as the nucleoside reverse transcriptase inhibitor (NRTI) alternatives to stavudine (d4T). The proportion of patients initiated after April 2010 when the treatment guideline changed was higher in the older cohort (27.2% vs. 20.4%, *p* < 0.05). The number of patients in the older cohort taking non-ART drugs associated with toxicity approached statistical significance (11.7% vs. 10.2%, *p* = 0.06). A lower proportion of the older cohort was taking non-ART drugs associated with a drug interaction (19.6% vs. 22.6%, *p* < 0.05).

**TABLE 1:** Baseline demographic, clinical, laboratory and regimen characteristics at the time of antiretroviral therapy initiation.

Variable	Age at ART initiation				PR (95% CI)
	18–39.9 years (*N* = 10726)		Over 50 years (*N* = 1635)		
	*n*	%	*n*	%
Male gender	3801	35.4	771	47.2	1.52 (1.39–1.66)
**Educational level**					
Secondary or higher	6749	84.5	602	48.5	0.24 (0.22–0.27)
Missing	2739	-	393	-	-
Unemployed	5878	54.8	860	52.6	0.93 (0.85–1.01)
**Alcohol use**					
Uses alcohol	1113	11.5	152	10.3	0.89 (0.76–1.05)
Missing	1070	-	156	-	-
**Smoking status**					
Smoker	936	9.7	191	12.9	1.32 (1.15–1.15)
Missing	1082	-	157	-	-
**Tuberculosis co-infection**					
Tuberculosis	1633	15.3	167	10.2	0.67 (0.57–0.78)
Missing	20	-	0	-	-
Other opportunistic infection	2502	23.3	276	16.9	0.70 (0.62–0.79)
Other AIDS-defining condition	162	1.5	29	1.8	1.15 (0.82–1.61)
WHO stage 3/4	4635	43.2	652	39.9	0.89 (0.81–0.97)
**Body mass index**					
< 18.5	1864	22.4	272	20.4	0.98 (0.86–1.12)
18.5–24.9	4797	57.6	715	53.6	-
≥ 125.0	1669	20.0	347	26.0	1.32 (1.18–1.49)
Missing	2396	-	301	-	-
**Haemoglobin**					
≤ 10.0 g/dL	2528	28.4	321	22.8	0.77 (0.69–0.87)
Missing	1812	-	230	-	-
**Alanine transaminase**					
> 40 U/L	1796	21.3	237	17.1	0.83 (0.73–0.95)
Missing	1859	-	249	-	-
**CD4+ count**					
0 cells/mm^3^ – 100 cells/mm^3^	5590	59.9	827	56.3	0.71 (0.54–0.93)
Missing	1389	-	165	-	-
**ART regimen**					
Contains nevirapine (vs. efavirenz)	1294	12.1	60	3.7	0.31 (0.24–0.40)
Contains zidovudine (vs. stavudine)	395	3.7	82	5.0	1.36 (1.11–1.66)
Contains tenofovir (vs. stavudine)	2106	19.6	361	22.1	1.16 (1.04–1.29)
Initiated after APR 2010 (vs. before APR 2010)†	2184	20.4	444	27.2	1.38 (1.25–1.53)
Non-ART drugs associated with toxicity‡	1097	10.2	192	11.7	1.14 (0.99)
Non-ART drugs associated with drug interaction‡	2422	22.6	320	19.6	0.85 (0.76–0.96)
Polypharmacy (> 5 non-ART medications) noted‡	6232	58.1	962	58.8	1.03 (0.94–1.13)

Data are presented as % (*n*) unless otherwise indicated.

Age median (IQR): 18–39.9 years = 32.8 (29.0–36.1); Over 50 years = 54.1 (51.8–57.6).

PR, prevalence ratio; CI, confidence interval; ART, antiretroviral therapy.

† , The South African National Department of Health ART guidelines changed on this date from first-line stavudine-containing regimens to first-line tenofovir-containing regimens;

‡ , These drugs are listed in the Appendix.

### Outcomes after 12 months of antiretroviral therapy

An overview of the treatment outcomes after 12 months of ART is provided in Table 2. A higher proportion of the older patients were dead (11.3% vs. 7.5%, *p* < 0.05) compared to the younger patients. There was no difference in LTFU. A higher proportion in the older group was transferred out (6.2% vs. 4.3%, *p* < 0.05). In the older group, proportionally fewer had a favourable response to treatment (54.1% vs. 63.8%, *p* < 0.05). This was because of a smaller number of patients having adequate CD4+ restoration (62.8% vs. 75.0%, *p* < 0.05); however, more older patients had a suppressed VL (89.5% vs. 86.5%, *p* < 0.05). The proportion of those with a treatment complication was similar in both age groups (54.6% vs. 53.9%, *p* = 0.66).

**TABLE 2:** Treatment status, immunological and virological response and treatment complications outcomes after 12 months of antiretroviral therapy by age at antiretroviral therapy initiation.

Treatment outcome	Age at ART initiation				PR (95*%* CI)
	18–39.9 years (*N* = 10726)		Over 50 years (*N* = 1635)		
	*n*	*%*	*n*	*%*
**Treatment status at 12 months**					
Alive and in care	8265	77.1	1189	72.7	Reference category
Dead	807	7.5	185	11.3	1.48 (1.29–1.71)
Lost to follow-up	1193	11.1	160	9.8	0.94 (0.81–1.10)
Transferred out	461	4.3	101	6.2	1.43 (1.19–1.72)
**Immunological response†**					
Increase CD4+ ≤ 100 cells/mm^3^	1584	25.0	347	37.2	Reference category
Increase CD4+ > 100 cells/mm^3^	4746	75.0	587	62.8	0.61 (0.54–0.69)
Missing CD4+	1935	-	255	-	
**Virological response**					
VL unsuppressed (≥ 400 copies/mL)	885	13.5	102	10.5	Reference category
VL suppressed (< 400 copies/mL)	5685	86.5	869	-	1.28 (1.05–1.56)
Missing VL	1695	-	218	89.5	
**Combined immunological and virological response‡**					
VL ≥ 400 and/or increase CD4+ ≤ 100 cells/mm^3^	2183	36.2	412	45.8	Reference category
VL < 400 copies/mL and increase CD4+ > 100	3840	63.8	486	54.1	0.71 (0.63–0.080)
Missing response	2242	-	291	-	
**Treatment complications†,‡**					
Absence of complications	3810	46.1	540	45.4	Reference category
Presence of complications	4455	53.9	649	54.6	1.02 (0.92–1.04)

PR, prevalence ratio; CI, confidence interval.

† , Based on patients alive and in care at 12 months;

‡ , Surrogate variables for treatment complications were regimen change, single drug switch, drug toxicity noted, VL at six months unsuppressed, missed medical appointment, missed drug pickup and low self-reported adherence.

### Multivariate analysis of associations with 12-month outcomes

An overview of the associations with 12-month outcomes in the older cohort is provided in Table 3. Baseline variables associated with an increased risk of death that persisted after multivariate analysis were age category of older than 55 years compared to age category of 50–55 years (PR 1.47, *p* < 0.05), the presence of another AIDS-defining condition (not an OI or TB) at initiation (PR 2.28, *p* < 0.05), baseline ALT > 40 U/L (PR 1.53, *p* < 0.05) and baseline CD4+ < 100 cells/mm^3^ (PR 2.15, *p* < 0.05). Initiation with an NVP-containing regimen approached significance (PR 2.04, *p* = 0.06) but the numbers on NVP were small. Factors associated with a favourable response to treatment that persisted after adjusted regression analysis were: being unemployed (PR 1.18, *p* < 0.05) and baseline ALT > 40 U/L, (PR 1.19, *p* < 0.05). Age older than 55 years approached statistical significance as being negatively associated with a favourable response to treatment (PR 0.87, *p* = 0.06). Associations with the presence of a treatment complication after multivariate analysis included: being unemployed (PR 1.12, *p* < 0.05), smoking at initiation (PR 1.20, *p* < 0.05) and taking an NVP-containing regimen (PR 1.36, *p* < 0.05). An education level of secondary school or higher (PR 0.87, *p* < 0.05) appeared to be protective.

**TABLE 3:** Adjusted baseline predictors of death, combined immunological and virological response, and presence of treatment complication at 12 months for subjects older than 50 years at antiretroviral therapy initiation.

Baseline predictor variable	Outcome		
	Death	Combined immunological and virological response‡	Presence of treatment complication§
	PR (95% CI)	PR (95% CI))	PR (95% CI)
Age at initiation > 55 years	1.47 (1.08–2.00)*	0.87 (0.76–1.00)	0.95 (0.85–1.06)
Education level secondary plus	0.91 (0.62–1.35)	1.09 (0.94–1.27)	0.87 (0.76–0.99)*
Unemployed	1.19 (0.87–1.65)	1.18 (1.03–1.33)*	1.12 (1.01–1.26)*
Alcohol user	1.08 (0.64–1.84)	1.16 (0.95–1.42)	1.12 (0.94–1.32)
Smoker	0.77 (0.45–1.36)	0.96 (0.77–1.21)	1.20 (1.03–1.40)*
**First regimen**			
Contains nevirapine	2.04 (0.97–4.31)	1.26 (0.84–1.89)	1.36 (1.07–1.72)*
Contains zidovudine	0.92 (0.37–2.31)	0.71 (0.43–1.19)	0.95 (0.67–1.34)
Contains tenofovir	0.68 (0.42–1.09)	1.09 (0.93–1.27)	0.91 (0.79–1.06)
Guideline period after April 2010	0.72 (0.47–1.11)	1.06 (0.91–1.24)	1.00 (0.88–1.14)
Non-ART drug associated with toxicity	0.96 (0.63–1.48)	0.96 (0.80–1.15)	0.96 (0.82–1.13)
Non-ART drug associated with drug interaction	0.94 (0.64–1.37)	0.95 (0.81–1.11)	0.95 (0.82–1.09)
Polypharmacy noted	1.26 (0.88–1.80)	1.04 (0.90–1.19)	1.00 (0.89–1.13)
Tuberculosis at initiation	0.71 (0.41–1.23)	0.92 (0.73–1.16)	0.95 (0.78–1.15)
Other opportunistic infection at initiation	0.80 (0.52–1.22)	1.05 (0.88–1.25)	1.00 (0.85–1.17)
Other AIDS-defining condition at initiation	2.28 (1.30–4.01)*	0.69 (0.33–1.40)	0.94 (0.61–1.47)
ALT > 40 U/L	1.53 (1.06–2.22)*	1.19 (1.02–1.40)*	0.98 (0.83–1.14)
CD4+ 0–100 cells/mm^3^	2.15 (1.48–3.13)*	1.08 (0.67–0.73)	0.85 (0.63–1.16)

PR, prevalence ratio; CI, confidence interval; ALT, alanine transaminase; ART, antiretroviral therapy.

* *p* ≤ 0.05

† , Adjusted for gender, baseline haemoglobin, baseline clinical stage of HIV infection as per the World Health Organization staging system and baseline body mass index;

‡ , VL < 400 copies/mL and increase CD4+ > 100 cells/mm^3^;

§ , Surrogate variables for treatment complications were regimen change, single drug switch, drug toxicity noted, VL at six months unsuppressed, missed medical appointment, missed drug pickup and low self-reported adherence.

## Discussion

In this study, we found that South African older adults initiated onto ART were a demographically and clinically distinct population with different outcomes when compared to younger adults. Despite better health markers at baseline and better virological suppression at 12 months after ART initiation, they had poorer immune reconstitution and higher mortality rates. They should be considered as a separate group within HIV treatment facilities for more specialised care as well as integrated chronic disease management.

The higher proportion of males in our study has been shown in other South African cohorts and mirrors prevalence data.^6,15^ Reasons for this were previously explored in a rural setting in Mpumalanga Province. These men were found to have riskier sexual practices such as multiple sexual partners, extramarital partners, low celibacy rates and low condom usage. There is an historical contextual component including factors such as migration for work leading to opportunities for infidelity; this was followed by back migration in later life and men then infecting their wives. Older men received pensions and may have had sexual relationships with younger women in return for financial reward (colloquially called ‘Blessers’). Older women reported lower libido and this resulted in men seeking younger partners. Findings such as these may help to direct preventative interventions.^25^ Men who have sex with men (MSM) could be contributing to the increased numbers; however, heterosexual spread still predominates in the South African setting.^9^

The lower education levels of the older group relate to the historical legacy of apartheid that resulted in the segregation of races under *the Bantu Education Act* of 1953. The curriculum for non-white people was of a lower standard, teachers were poorly paid and unqualified, resources were comparatively limited and education was not free. This lasted from 1954 until 1980.

The increased proportion of smokers is important to note, as smoking has multiple health implications in HIV-positive people including increased vascular and respiratory illnesses and higher associated mortality. In our study, smoking was associated with the presence of a treatment complication.^26^ Assistance with smoking cessation could be an important part of health promotion in this age group; however, the prevalence of smoking in our cohort was still lower than the national average.^27^

The older group had a lower prevalence of TB, OIs and advanced WHO stage disease at baseline. There was a lower proportion in the CD4+ count 0 cells/mm^3^ – 100 cells/mm^3^ category. They had a higher proportion in the BMI category > 25 kg/m^2^. This seems to indicate better health status at initiation and is contrary to resource-rich settings where studies show that older HIV-infected adults presented with more advanced disease.^15^ Outcomes in terms of mortality and immunological response to treatment were worse in older patients than their younger counterparts. This has also been shown in other South African studies. A large South African multicentre study of the Kheth’Impilo cohort conducted across both urban and rural areas showed that a lower proportion of older patients (in this study > 55 years) had CD4+ counts of < 50 cells/mm^3^, WHO stage 4 disease and TB treatment at baseline. They also showed that viral suppression was greater in older patients compared to younger patients and that the CD4+ response was slower and lower in the older cohort with increased mortality in the older cohort.^4^ However, despite better baseline health markers and better virological suppression, older age results in poorer treatment outcomes. This phenomenon may only be in the short term – an effect not looked at in our study. In the Hlabisa HIV Treatment and Care Programme in Kwazulu-Natal, older age was significantly linked to higher early mortality (particularly in the first three months of treatment) in the face of higher initiation CD4+ counts. However, after 12 months of treatment, this difference was no longer noted regardless of CD4+ response. They also found better virological response to ART but poorer immunological responses compared to younger adults.^15^ The authors of that study proposed that the levelling out of mortality after 12 months might represent survivor bias, better virological response to ART and improved access to healthcare for other chronic illnesses.^15^

The increased number of older patients initiated after 2010 compared to before reflects the trend of increased older persons with being diagnosed with HIV.^6^

HIV-associated non-AIDS comorbidities such as liver disease, cardiovascular disease, kidney dysfunction, osteoporosis, cognitive impairment, frailty and non-AIDS cancers are common in older HIV-infected adults. Although HIV is known to exacerbate chronic illnesses and chronic inflammation, in a rural South African community, access to general chronic illness screening for HIV-positive patients attending clinics established for HIV treatment may have actually reduced morbidity because of regular contact with healthcare services that they would otherwise not have.^28^ Ideally, older HIV-infected adults should be screened for other age-related chronic illnesses as part of holistic HIV care. Services should be integrated to avoid the need to attend several separate clinics for treatment of other chronic illnesses.

In our study, a higher proportion in the older cohort were taking drugs associated with drug toxicity, and there were similarly high rates of polypharmacy across the two groups. Despite this, treatment complications were not increased. This contradicts international data but correlates with other South African studies.^13^ Loss to follow-up was lower and viral rebound, and a regimen switch was less likely in older patients in a South African multicohort public sector study.^4^ This suggests that despite increased likelihood of comorbid conditions, cognitive impairment and multiple other medications, older South African patients are compliant with and tolerate standard ART regimens with a lower risk of non-adherence and treatment is successful.

LTFU rates were no different between the two groups, and TLC has active tracing methods for patients that are LTFU and also the ability to check against South African vital registries in order to avoid underestimating mortality, a technique not used in other similar studies. This improved the accuracy of the outcome status of dead.^4,24^

Within the older cohort, age ≥ 55 years was associated with death. Unsurprisingly, very low CD4+ count at initiation as well as the presence of an AIDS-defining condition was associated with increased risk of death. Baseline elevation of ALT was also associated with death, and liver disease has a poor prognosis in HIV-infected patients.^29^ The almost significant association of the drug NVP with death should be interpreted cautiously as the numbers on NVP were extremely small. In the absence of childbearing potential, NVP was generally used for persons with cognitive or psychiatric illness or night shift workers. This may have caused a selection bias. In a similar study, NVP was associated with increased likelihood of a regimen switch.^4^ Unfortunately, renal dysfunction was not analysable because of a large amount of missing data. This would have been relevant in view of the use of TDF and a high proportion of baseline renal dysfunction among older HIV-infected adults in other studies such as the Hlabisa cohort.^15^

Unemployment was associated with a favourable response to treatment and this seems surprising. The majority of the older group was under 60 years, that is, below the age of eligibility for old age pensions. However, they may have had access to disability pensions, and unemployment is a prerequisite to qualify for these benefits. The association of increased ALT levels with a favourable response to treatment is also counter-intuitive (increased baseline ALT was also associated with a higher risk of death) but is perhaps explained by more frequent clinical monitoring and favouring or avoiding certain medication regimens in the presence of liver dysfunction (e.g. NVP avoidance in liver disease). Age of 55 years and above showed a negative association with a favourable treatment outcome in keeping with previous research.^4^

Higher education levels were protective against treatment complications. This may have been because of improved understanding of the treatment and better access to healthcare. The presence of a treatment complication was associated with unemployment and smoking. Unemployment could represent a lower functional status. Smoking has previously been shown to affect ART efficacy adversely.^27^ NVP use was also associated with a treatment complication. This association has been described in other studies and warrants further examination.^4,27^

Our study has several limitations including large amounts of missing data, in particular non-ART medication as well as laboratory data. Non-ART drugs and comorbidities may not have been accurately captured because of operational issues as the HIV clinic and ART pharmacy does not record or supply many of the medications for other chronic medical conditions, and these are prescribed and dispensed through a separate system. Unfortunately, because of the large amount missing and concern regarding selection bias, renal function in terms of glomerular filtration rate data was non-interpretable. This would have been highly relevant in the context of older adults taking TDF. We did not have access to a functional score, cognitive test or frailty marker – all clinically pertinent in older patients in view of the heterogeneity of health. This clinic serves a broad population of HIV-positive people and is not specifically geared to older adults. However, we hope that our study creates the impetus to change this. Although this is a large study undertaken in a real-life setting, it only encompasses one centre and its findings may not be applicable to those that are not urban-dwelling South Africans. The time frame of 12 months is a further limitation as the CD4+ restitution may have been further improved over a longer time period. We did not analyse early (three months) and later outcomes as has previously been done, and this may have given additional information.^15^ The change in guideline and extension of eligibility from a CD4+ of < 200 cells/mm^3^ to < 350 cells/mm^3^ may have affected outcomes and subgroups were not analysed; however, only a small minority of patients were initiated at a CD4+ count of > 200 cells/mm^3^ (194/1.8% of the younger cohort vs. 43/2.62% of the older cohort) across the study period so the expanded rollout did not appear to have made a difference to when the patients presented. This is supported by a recent paper showing a high proportion of late stage presentation over various guideline eras in South Africa despite easier access to ART – most people appear to present when they are sick.^30^

## Conclusion

It is important to consider the unique characteristics of the increasing numbers of HIV-positive older adults in South Africa. Clinicians need to maintain a high index of suspicion and test for HIV in this age group. Sexual risk campaigns around HIV prevention should be tailored to this older age group, particularly men. Presenting to healthcare for HIV treatment should be used as an opportunity to screen and treat for smoking, obesity and other non-communicable diseases within integrated health services. Antiretroviral therapy initiation in the older person should be expedited in view of higher mortality despite a clinically well patient and slower immune restitution and the move towards ‘test and treat’ in South Africa is likely to benefit this older group provided the diagnosis is made early. The use of NVP in older patients might require caution and further study. Closer monitoring of treatment is advised. An awareness of polypharmacy and non-ART medications associated with toxicity or drug interactions is important to reduce potential adverse effects.

Compliance and VL suppression is as good in the older age group but CD4+ response is slower or attenuated. Older adults have different characteristics and needs. Special guidelines should be considered.
